# Crosstalk between ferroptosis and steroid hormone signaling in gynecologic cancers

**DOI:** 10.3389/fmolb.2023.1223493

**Published:** 2023-07-04

**Authors:** Wen Lai, Jianquan Chen, Tianming Wang, Qiaoling Liu

**Affiliations:** ^1^ Department of Obstetrics and Gynecology, The Affiliated Jiangning Hospital with Nanjing Medical University, Nanjing, China; ^2^ Central Laboratory, Translational Medicine Research Center, The Affiliated Jiangning Hospital with Nanjing Medical University, Nanjing, China

**Keywords:** ferroptosis, lipid peroxidation, gynecologic cancers, estrogen, progesterone

## Abstract

Ferroptosis is a novel types of regulated cell death and is widely studied in cancers and many other diseases in recent years. It is characterized by iron accumulation and intense lipid peroxidation that ultimately inducing oxidative damage. So far, signaling pathways related to ferroptosis are involved in all aspects of determining cell fate, including oxidative phosphorylation, metal-ion transport, energy metabolism and cholesterol synthesis progress, et al. Recently, accumulated studies have demonstrated that ferroptosis is associated with gynecological oncology related to steroid hormone signaling. This review trends to summarize the mechanisms and applications of ferroptosis in cancers related to estrogen and progesterone, which is expected to provide a theoretical basis for the prevention and treatment of gynecologic cancers.

## 1 Introduction

**FIGURE 1:**
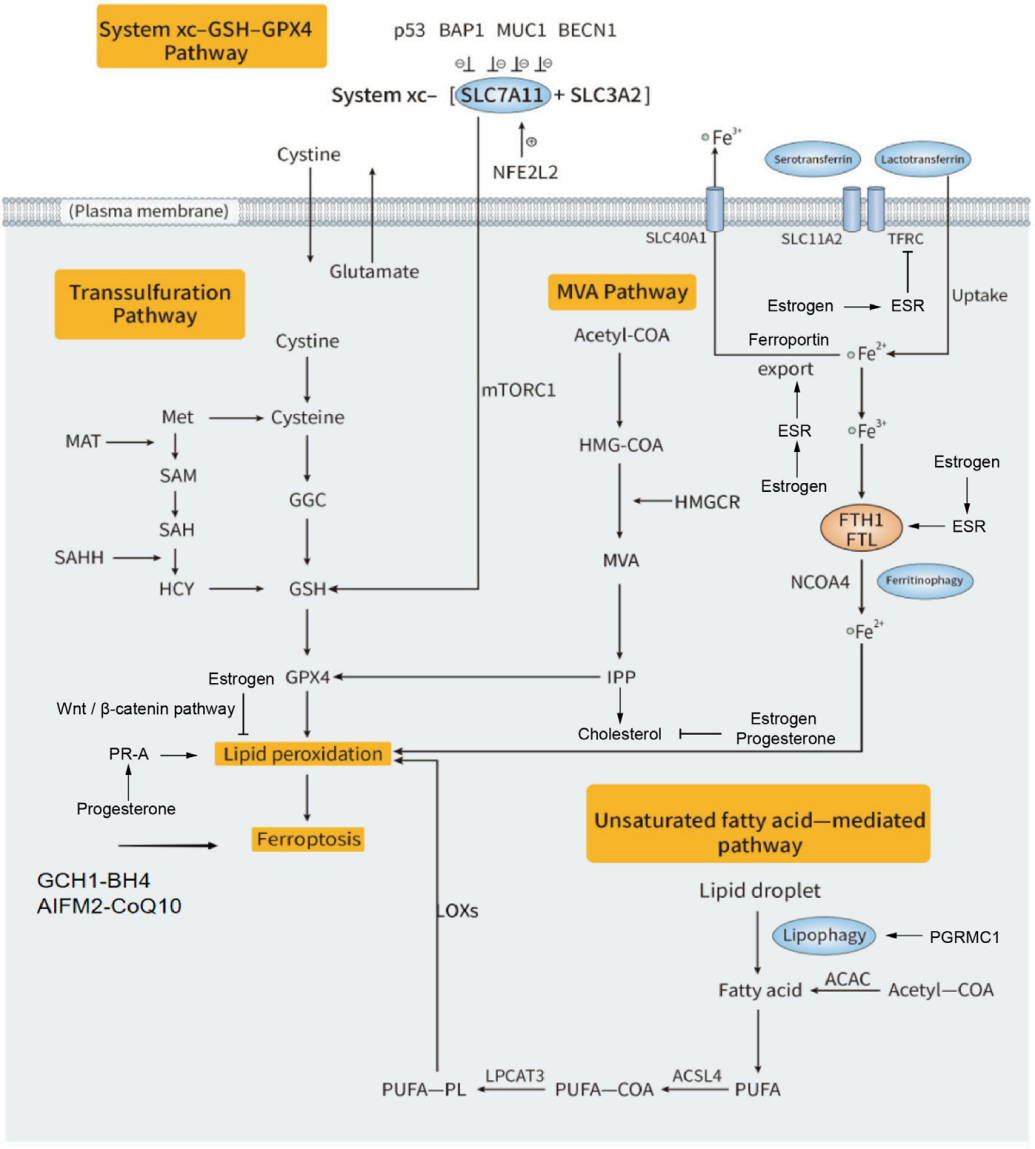
Regulation mechanisms of estrogen and progesterone on ferroptosis in gynecologic cancers. Ferroptosis is driven by iron-dependent lipid peroxidation. There are multiple molecular mechanisms involved in the regulation of ferroptosis. Iron-loaded TF-TFRC complexes releases Fe^2+^ into the cytoplasm via SLC11A2. The intracellular free iron is stored as Fe^3+^-ferritin complex. The autophagy-dependent ferritinophagy is mediated by NCOA4 for degradation of ferritin at lysosome to release Fe^2+^. The excess Fe^2+^ induces lipid peroxidation via Fenton reaction. PUFAs, which are mainly from ACAC-mediated fatty acid synthesis or by the lipophagy, also can induce ferroptosis. PUFAs convert to PUFA-PL by ACSL4 and LPCAT3, finally induce lipid peroxidation. The system xc−-GSH-GPX4 pathway mainly acts as a defense system so as to antiferroptosis. In this pathway, cystine enters the cell and is oxidized to cysteine, with the action of GCL and GSS, GSH is synthesized and catalyzed by GPX4 to antiferroptosis. IPP, a metabolic intermediate from MVA pathway related to cholesterol synthesis, can enhance GPX4 activity and cause antiferroptotic effect. Transsulfuration pathway, GCH1-BH4 pathway and AIFM 2-CoQ10 pathway also have unique mechanisms to antiferroptosis. Estrogen binds to ESR and reduces free iron by inhibition of TFRC and ferritin or by promotion of ferroportin. Estrogen can also suppress lipid peroxidation through Wnt/β-catenin pathway. Progesterone enhances lipid peroxidation via its receptor PR-A. The other progesterone receptor PGRMC1 also mediated lipophagy to increase ROS generation. Both estrogen and progesterone supplement can inhibit cholesterol synthesis, which regulates GPX4 in turn.

Ferroptosis is a unique iron-dependent non-apoptotic cell death and is considered as one of the most widely studied regulated cell death types in the last decade. The concept of ferroptosis is first proposed by Dixon et al., in 2012, they triggered ferroptosis with erastin (a selective lethal small molecule drug targeting the oncogenic *RAS*) and described its major features (Dixon et al., 2012). During ferroptosis, the outer mitochondrial membrane is ruptured and the mitochondrial cristae reduction is commonly observed in mitochondrial morphology. The regulation of ferroptosis is associated with iron homeostasis and lipid metabolism (Li et al., 2020). Multiple factors caused Fe^2+^ accumulation that generates numerous reactive oxygen species (ROS) from hydrogen peroxide through the Fenton reaction and lead to ferroptosis. Lipid peroxidation is considered as the primary driver of ferroptosis (Gaschler and Stockwell, 2017; Kagan et al., 2017). Either the depletion of glutathione (GSH) or reduction of glutathione peroxidase 4 (GPX4) activity would attenuate lipid peroxide metabolism, increase ROS level and cause ferroptosis. The molecular mechanism of ferroptosis is involved in a complex regulation network such as system xc−-GSH-GPX4 pathway, serotransferrin-mediated iron uptake, unsaturated fatty acid-mediated lipid peroxidation, and cholesterol synthesis related mevalonate pathway, et al. (Figure 1). We are interested in the crosstalk between ferroptosis and steroid hormone signaling pathway in gynecologic cancers. Here this review trends to summarize the molecular mechanisms of ferroptosis on steroid hormone signaling pathway and its applications in gynecologic cancers.

## 2 Molecular mechanism of ferroptosis

Ferroptosis occurs mainly by targeting two pathways (extrinsic and intrinsic pathways) (Tang and Kroemer, 2020). In the extrinsic pathway, ferroptosis begins with the inhibition of cystine/glutamic acid transporter (system xc−) or with the activity of the serotransferrin (TF)-mediated iron uptake. In the intrinsic pathway, it is activated by blocking intracellular antioxidant enzyme such as GPX 4 (Chen et al., 2021a).

### 2.1 System xc−-GSH-GPX4 pathway

System xc−-GSH-GPX4 pathway is the main defense system to antiferroptosis. The system xc− is comprised of two subunits: solute carrier family 7 member 11(SLC7A11) and solute carrier family 3 members 2 (SLC3A2) (Sato et al., 2000). After the exchange of Cysteine and Cystine, system xc− maintains the GSH generation in a continuous reactions (Lewerenz et al., 2013). In mammalian cells, one of the most important functions of system xc− is mediating Cystine transport by glutamate reverse transportation. Cystine is convert to cysteine, then cysteine is catalyzed by glutamate-cysteine ligase (GCL) and glutathione synthetase (GSS) for GSH synthesis (Bayır et al., 2020). SLC7A11 is commonly used as the target of system xc−. SLC7A11 promotes the expression of GPX4 through the mTORC-4EBP1 signaling pathway (Zhang YY. et al., 2021). Up-regulating the expression of system xc− is involved in the enhanced chemoresistance and tumor growth (Ishimoto et al., 2011; Habib et al., 2015). Inhibiting SLC7A11 causes GSH depletion (Dixon et al., 2012). Down-regulating system xc− by targeting TP53 (Jiang et al., 2015; Liu S. et al., 2022), NFE2L2 (Chen et al., 2017), BAP1 (Zhang et al., 2018), BECN1 or OTUB1(Song et al., 2018), can reduce GSH synthesis, enhance ROS generation, and result in ferroptosis. GPX4 belongs to GSH peroxidases and it is an antiferroptotic molecular (Seibt et al., 2019). It reduces the generation of phospholipid hydroperoxide and converts it to phospholipid alcohol. The GPX4 activity is depended on the presence of GSH and selenium, which finally affect ferroptosis (Ingold et al., 2018). Some small molecular compounds can inhibit GPX4 activity directly or indirectly to induce ferroptosis, and some other compounds can lead to the degradation of GPX4 (Yang et al., 2014; Shimada et al., 2016). High expression level of GPX4 is correlated with bad prognosis in breast cancer (BC) patients, and the GPX4 reduction enhances the sensitivity of cancer cells to cisplatin (Zhang et al., 2020).

### 2.2 Serotransferrin-mediated iron uptake

Intracellular Fe^2+^ accumulation is one of central events to induce ferroptosis. The increased iron uptake and the reduced iron storage as well as the limited iron efflux can induce ferroptosis (Chen X. et al., 2020). Iron metabolism capability determines cell susceptibility to ferroptosis by regulating cell labile iron (LIP). Increasing LIP could enhance the Fenton reaction so as to produce more hydroxyl radicals (Feng et al., 2020). The iron-loaded Serotransferrin (TF) binds to Transferrin receptor protein 1 (TFRC) locating in cytomembrane and forms a TF-TFRC complex (Yang and Stockwell, 2008; Wang Y. et al., 2020). The complex releases iron (Fe^2+^) into the cytoplasm mediated by solute carrier family 11 member 2 (SLC11A2) (Montalbetti et al., 2013; Gao et al., 2015). The intracellular free iron is stored as a ferritin-Fe^3+^ complex (Muhoberac and Vidal, 2019) and this complex releases Fe^2+^ through a ferritinophagy manner (Park and Chung, 2019). Ferritinophagy is an autophagy-dependent degradation of ferritin progress. During ferritinophagy, the ferritin-Fe^3+^ complex is mediated by nuclear receptor coactivator 4 (NCOA4) to degrade in autolysosome and releases Fe^2+^, which increases cell sensitivity to ferroptosis (Mancias et al., 2014; Mancias et al., 2015; Gatica et al., 2018; Liang et al., 2022). Enhancing iron output or increasing ferritin output can suppress ferritinophagy. The iron output is mediated by solute carrier family 40 member 1 (SLC40A1) in cytomembrane, the ferritin output is mediated by exosome, both of them are able to inhibit ferroptosis (Geng et al., 2018; Brown et al., 2019).

### 2.3 Unsaturated fatty acid-mediated lipid peroxidation

The unsaturated fatty acids-mediated lipid peroxidation is an important pathway to induce lipid peroxidation and ferroptosis. The intracellular free fatty acids are mainly generated from two ways: the first way is the fatty acid *de novo* synthesis mediated by Acetyl-CoA carboxylase (ACAC); the second way is the fatty acid release derived from lipid droplet (LD). In the fatty acid *de novo* synthesis progress, acetyl-CoA is catalyzed by ACAC to malonyl-CoA, and then is subjected to a continuous polymerization into fatty acids (Batchuluun et al., 2022). In this progress, several major enzymes (including ACLY, ACSs, ACC, FASN, and SCD1) are involved in fatty acid generation (Li et al., 2022). The excess free fatty acids are usually synthesized into triacylglycerols (TAGs), which are mainly stored in LD. Multiple enzymes regulate LD formation and LD catabolism: during LD formation, GPAT, AGPAT, Lipin and DGAT are required (Tan et al., 2014; Onal et al., 2017); during LD catabolism, ATGL, HSL and MGL are the rate-limiting enzymes of lipolysis that catalyze LD into fatty acids step-by-step (Zechner et al., 2012; Missaglia et al., 2019; Wang T. et al., 2020); in addition to lipolysis, an autophagy-dependent progress referred to lipophagy also has a function of LD breakdown mediated by acid lipases in the autolysosome (Kaur and Debnath, 2015; Schott et al., 2019). The polyunsaturated fatty acids (PUFAs), either from ACAC-mediated *de novo* synthesis or LD breakdown, are able to trigger ferroptosis. In this progress, long-chain fatty acid–CoA ligase 4 (ACSL4) and lysophospholipid acyltransferase 5 (LPCAT3) are required. Combining with CoA, ACSL4 catalyzes the free arachidonic acid (AA) or adrenergic acid (Ada), which is most likely to undergo peroxidation, to form AA-CoA or Ada-CoA (Yuan et al., 2016; Doll et al., 2017; Kagan et al., 2017). LPCAT3 promotes PUFA-CoA and phospholipid (PL) to form into PUFA-PL, enhances lipid peroxidation and induces ferroptosis (Dixon et al., 2015; Chen et al., 2021a).

### 2.4 Mevalonate (MVA) pathway

Cholesterol can be produced by receptor-mediated LDL-cholesterol uptake or cholesterol *de novo* synthesis. In cholesterol biosynthesis pathway, three molecules of acetyl-CoA are catalyzed by HMG-CoA synthase into 3-hydroxy-3-methylglutaryl-CoA (HMG-CoA). This CoA-derivative is then converted to Mevalonate (MVA) through a reduction reaction by HMG-CoA reductase (HMGCR), which is a rate-limiting enzyme for cholesterol biosynthesis (Liao et al., 2016; Ni et al., 2021). In the next step, MVA forms isopentenyl diphosphate (IPP) via phosphorylation and decarboxylation (Kuzuyama and Seto, 2012; Bathaie et al., 2017). With catalytic action of the endoplasmic reticulum cyclase and oxygenase, IPP is catalyzed into squalene, then into lanosterol, and is finally converted to cholesterol through multiple steps. In addition, IPP also has a function of antiferroptosis, it promotes the expression of GPX4 to defend lipid peroxidation so as to inhibit ferroptosis (Warner et al., 2000; Ingold et al., 2018). Statins can also reduce GPX4 and inhibit ferroptosis by targeting HMGCR activity through MVA pathway (Yu et al., 2017).

### 2.5 Other pathways

Besides the system xc−, cysteine can also be produced through the thiolation. The transsulfuration pathway is another antioxidant process that inhibits lipid peroxidation. In this pathway, methionine is converted by methionine adenosyltransferase to S-adenosylmethionine and is further converted to S-adenosylhomocysteine (SAH). The S-adenosylhomocysteine hydrolase (SAHH) hydrolyzed SAH to the cysteine precursor homocysteine (HCY) (Chen et al., 2016). It is reported that increasing DJ-1 (an oxidative stress-related protein) promotes the stability of SAHH activity and HCY synthesis through this pathway (Cao et al., 2020). Knockdown of Cysteine-tRNA synthetases (CARS), which is a molecular that links cysteine to tRNA for protein translation, promotes transsulfuration, enhances cysteine synthesis, increases GSH, and inhibits ferroptosis (Yao and Fox, 2013; Hayano et al., 2016).

In addition, Apoptosis-inducing factor mitochondrion-associated 2 (AIFM2)-Coenzyme Q10 (CoQ10) axis is another antiferroptosis pathway. AIFM2 (also known as FSP1) is a NADP-dependent oxidoreductase of CoQ10 (Wei et al., 2020). CoQ10 is a lipophilic compound and is considered as a lipophilic free radical scavenger. It is reported that overexpressing AIFM2 inhibited ferroptosis and positively correlated with ferroptosis resistance in many cancers (Bersuker et al., 2019).

Similarly, the GCH1-BH4 pathway is reported to inhibit lipid peroxidation and defend ferroptosis (Kraft et al., 2020). Tetrahydrobiopterin (BH4) is an integral part of the antioxidant system and GTP cyclohydrolase-1 (GCH1) is the rate-limiting enzyme in the synthesis of BH4 (Latremoliere and Costigan, 2011; Cronin et al., 2018). GCH1 selectively inhibited the peroxidation of certain PUFA-PL, overexpressing GCH1 rescues ferroptosis induced by RSL-3 (a GPX4 inhibitor) in mouse fibroblasts.

## 3 Steroid hormone and steroid hormone signaling

### 3.1 Steroid hormone synthesis

**FIGURE 2:**
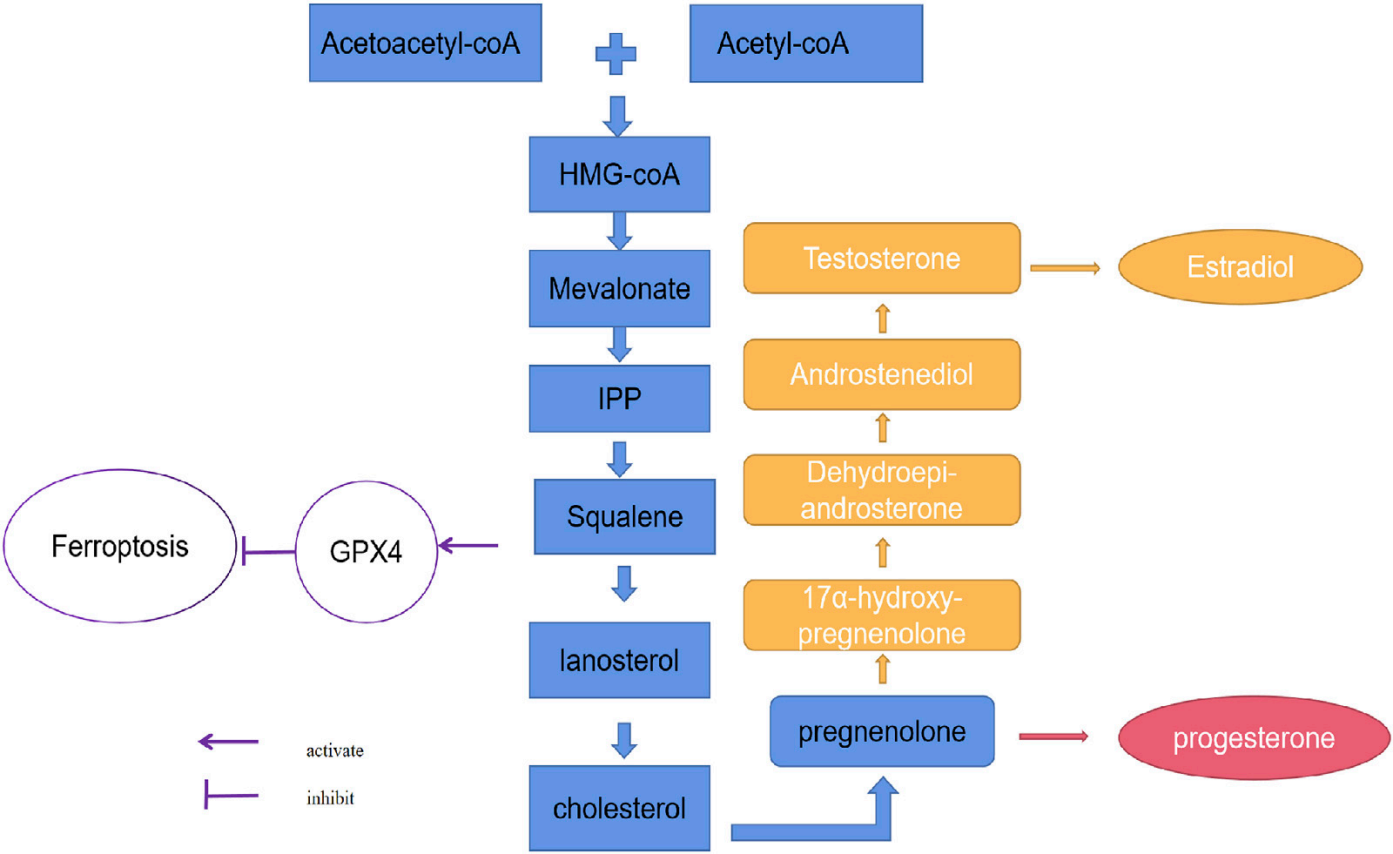
The relationship between ferroptosis and the synthesis of estrogen and progesterone. The synthesis of estrogen and progesterone depends on cholesterol. Three molecules of acetyl-CoA condense successively to form HMG-CoA. HMG-CoA is then reduced to MVA by HMGCR, and is further phosphorylated and decarboxylated to form IPP. IPP is condensed to produce squalene and then squalene forms lanosterol by the catalysis of endoplasmic reticulum cyclase and oxygenase, finally lanosterol is converted to cholesterol. Cholesterol enters into mitochondria and converts to pregnenolone by P450scc. Then 17-OH pregnenolone is converted to DHEA and finally forms estrogen.

It is well known that the main substrate for estrogen and progesterone synthesis is cholesterol. The receptor-mediated LDL-cholesterol uptake and cholesterol *de novo* synthesis is the major source of cholesterol. In the progress of receptor-mediated LDL-cholesterol uptake, after endocytosis, the cholesterol ester is cleaved by acid lipase in lysosome, free cholesterol is then transferred onto NPC intracellular cholesterol transporter 1 (NPC1) which is located in lysosomal membrane, and followed by a further transport to other organelles (Pfeffer, 2019). Cholesterol also could be generated through cholesterol *de novo* synthesis pathway. In this pathway, HMG-CoA, which is condensed from acetyl-CoA, is then reduced to MVA by HMGCR, and is further phosphorylated and decarboxylated into IPP. IPP is used to generate cholesterol through multiple steps (Kuzuyama and Seto, 2012; Liao et al., 2016; Bathaie et al., 2017; Ni et al., 2021). Then, as for substrate of steroid hormone synthesis, cholesterol could be catalyzed into estrogen and progesterone. Cholesterol enters into mitochondria, it is then catalyzed to pregnenolone by a cytochrome P450 monooxygenase referred to Cholesterol side-chain cleavage enzyme (CYP11A1 or P450scc) through hydroxylation of the side-chain and cleavage (Strushkevich et al., 2011). Pregnenolone or progesterone could be hydroxylated by Steroid 17-alpha-hydroxylase/17,20 lyase (CYP17A1 or P450c17) to form 17-alpha hydroxy metabolites. Then 17-OH pregnenolone is converted to dehydroepiandrosterone (DHEA) and finally forms estrogens (Auchus et al., 1998; Miller, 2002; DeVore and Scott, 2012; Petrunak et al., 2014; Yoshimoto et al., 2016) (Figure 2). Steroid hormones also have the capability to regulate intracellular cholesterol level in turn. A previously study referred to the effect of steroid hormone on cholesterol synthesis, has demonstrated that the inhibition of cholesterol synthesis could be found in the treatment of many steroids (DHEA, beta-estradiol, pregnenolone, progesterone and deoxycorticosterone included) (Metherall et al., 1996).

### 3.2 Steroid hormone signaling pathway

Estrogen is mainly generated by granulosa cells of ovaries (Fuentes and Silveyra, 2019). It maintains normal physiological function such as reproductive system development, body metabolism level regulation, immune regulation and a variety of sex hormone-driven cancers (Baker et al., 2017; Kumar and Goyal, 2021). Estrogen encompasses four estrogenic steroid hormones (estrone, 17-beta-estradiol (E_2_), estriol and estetrol). Among the four terms, E_2_ has the highest affinity of estrogen receptors (ERs) (Russell et al., 2019). ERs are divided into two categories: nuclear receptor type and membranous receptor type. The nuclear receptors mainly contain ESR1 (also called ERα) and ESR2 (also named ERβ), while the membranous receptor is G protein-coupled estrogen receptor (GPER1). ESR1 and ESR2 are highly homologous in the amino acid sequence of their DNA binding domain (96%) and the ligand binding domain (55%) (Kuiper and Gustafsson, 1997). ESR1 locates in epithelial and muscle cells of the uterus and vagina, epithelial and stromal cells of the breast, germinal epithelium of the ovary, and testicular interstitial cells (Pelletier and El-Alfy, 2000). ESR2 has a broad distribution of tissues, including the gastrointestinal tract, lung, and brain (Younes and Homma, 2011). They trigger different transcriptional responses and have opposite effects to determine the cell fate (Kwon et al., 2020). In the genomic effects of estrogen-mediated signaling, estrogen binds to ESR1 or ESR2, forms a estrogen-receptor complex, and regulates the transcription of downstream genes in the nucleus by interaction with estrogen response elements (EREs) directly or by tethering to Sp-1 and Ap-1 (Fuentes and Silveyra, 2019; Chen P. et al., 2022). Under physiological condition, estrogen regulates a variety of cellular processes such as autophagy, proliferation, apoptosis, survival, differentiation, and vasodilation. It also regulates Ca^2+^ mobilization, PI3K signaling, and MAPK pathway through membrane-bound ERs with a non-genomic effect (Chen YC. et al., 2022).

In addition to estrogen, progesterone also plays an important role in gynecologic cancers (Kim et al., 2013). Progesterone is a natural progestin, it is produced from follicular granulosa cells after reaching the luteinizing hormone (LH) peak in the middle of the menstrual cycle, and is mainly produced by the corpus luteum and placenta (Bulletti et al., 2022). Besides the functions of maintaining the implantation of embryonic endometrium and sustain pregnancy, progesterone has multiple biological effects such as utero-relaxation and neuroprotection (Bulletti et al., 1993; Jodhka et al., 2009). In animal models, progesterone affects cognitive ability and suggests its potential role of cognitive ability in human (particularly more relevant for women) (Henderson, 2018). Classical progesterone signaling pathway can be activated by the steroid hormone progesterone through nuclear progesterone receptor (nPR), which has two major isoforms (PR-A and PR-B) (Ali et al., 2023). PR-A is necessary for uterine development and PR-B is necessary for mammary gland development (Conneely and Lydon, 2000). The two isoforms can form a homodimer or heterodimer, and regulate the transcription of downstream genes in the nucleus by interaction with progesterone response elements directly or by tethering to Sp-1 and Ap-1 (Tsai et al., 1988; Tseng et al., 2003; Daniel et al., 2011). Progesterone also activates non-classical pathway via non-nuclear PR containing progesterone receptor membrane component 1 (PGRMC1) and progesterone receptor membrane component 2 (PGRMC2). Numerous studies in endometriosis (EMs) have shown the association of progesterone and iron overload (Van Langendonckt et al., 2002; Van Langendonckt et al., 2004; Lousse et al., 2009). In addition, progesterone takes part in ferroptosis by affecting protein-protein interaction. Progesterone targets Fibulin-1 (FBLN1) in the process of endometrial stromal cells (ESCs) decidualization, FBLN1 interacts with EGF-containing fibrin-like extracellular matrix protein 1 (EFEMP1) and affects the stability of EFEMP1. Silencing of *EFEMP1* inhibits the effect of FBLN1 on ferroptosis (Wan et al., 2022).

## 4 Crosstalk in gynecologic cancers

### 4.1 Steroid hormone signaling and gynecologic cancers

Ovarian cancer (OVCA) is one of the most lethal malignancies, including epithelial tumor, sexual cord-mesenchymal tumor and germ cell tumor. The epithelial OVCA is most common among all the OVCA types with less than 50% on the 5-year relative survival rate (Torre et al., 2018). High estrogen level is often observed in patients with OVCA, and the estrogen receptor is also high expressed in OVCAs (Mungenast and Thalhammer, 2014; Englert-Golon et al., 2021; Xu et al., 2022). High progesterone level also increases the risk of OVCA. The progesterone signaling promotes the development of high-grade serous carcinoma into metastatic OVCA via BRCA1/DNA repair signaling pathway (Kim et al., 2020).

Endometrial cancer (EC) is the sixth most common cancer in the world. In the United States, its incidence rate increases year by year. The prognosis of patients with recurrent EC and clinical histological detection of aggressive EC is usually limited (Siegel et al., 2019). Surgery, radiotherapy and chemotherapy are the current approach for EC treatment, but have unsatisfactory effects. Estrogen stimulates EC proliferation, while progesterone inhibits it. In estrogen-dependent EC, ESR1 is high expressed, it interacts with GPER and promotes the proliferation of EC cells via PI3K signaling pathway and MAPK signaling pathway (Yu et al., 2022). Progesterone acts to antagonize estrogen in EC. PR is mainly expressed in epithelial cells and stromal cells. It inhibits the proliferation of EC cells with a paracrine manner (Kim et al., 2013; Gompel, 2020).

Cervical cancer (CC) is also most common in women, and almost all cases of cervical squamous cell carcinoma can be attributed to the infection of human papillomavirus (HPV). Basing on their carcinogenic potential, HPVs are classified as low-risk HPV (lrHPV) or high-risk HPV (hrHPV) (Bedell et al., 2020). About 67% of HPV infections will be eliminated within 12 months without intervention and the eliminated rate would reach 90% within 24 months, however, the remaining HPV infections might have high persistent potential (Plummer et al., 2007). Persistent HPV infection will lead to an abnormal proliferation in the lesion region named cervical intraepithelial neoepithelial neoplasia (CIN). According to pathological grade, CIN is classified as CINI, II, and III, or is classified as low-grade squamous intraepithelial lesion (LSIL) and high-grade squamous intraepithelial lesion (HSIL). CINI corresponds to LSIL or dysplasia, CINII and most CINII correspond to HSIL or moderate and severe dysplasia (Jayshree, 2021). CC is a non-estrogen-dependent cancer. But estrogen coordinates with HPV by increasing the DNA double-strand breaks (DSBs), then it promotes proliferation of CC cells through G protein-coupled receptor 30 (GPR30) signaling pathway (Ogawa et al., 2023). The estrogen receptor ESR1 is expressed higher in the nucleus of squamous epithelium than that in cervical lesions. And the expression of ESR1 in squamous epithelium is higher than that in the cervical glands. Progesterone receptor is important for suppression of CC occurrence, low expression of PR may increase the risk of CC, activating PR by medroxyprogesterone acetate reduces the occurrence of CC (Park et al., 2021; Baik et al., 2022). Notably, PGRMC1 has been reported to promote migration of CC cells, siRNA-mediated *PGRMC1* knockdown reduces the proliferation of CC cells and inhibits the migration ability of CC cells (Shih et al., 2019).

Besides to gynecologic cancers, breast cancer (BC) is considered to be the most relative cancer of steroid hormone signaling. BC is one of the highest incidence cancers in the world (Duggan et al., 2021). The breast has a unique microenvironment containing a large number of adipocytes. Thus, hormone signaling and lipid metabolism capability may be involved in the invasion and metastasis of BC. In hormone-dependent BC, estrogen has a high level in BC and it can induce DNA damage by metabolites (Starek-Świechowicz et al., 2021). Estrogen activates PI3K signaling pathway by ERs to promote cell proliferation of BC cells where ERs is highly expressed (Vasan et al., 2019). Progesterone influences early events in the occurrence of BC. PR-B mediates the proliferation of BC cells related to progesterone and it also regulates the actions of extranuclear signaling of PRs (Trabert et al., 2020).

### 4.2 Steroid hormone signaling and ferroptosis

The relation between ferroptosis and steroid hormone signaling has been studied in a variety of cancer types from molecular mechanism to the development of targeted therapeutic drug. Activation of estrogen-related receptor gamma (ESRRG) may enhance the effect of ferroptosis in HCC cells with sorafenib-resistant (Kim et al., 2022). Increasing PGRMC1 expression promotes fatty acid oxidation and enhances the sensitivity of paclitaxel-tolerant cancer cells (PCC) to ferroptosis in head and neck cancer (You JH. et al., 2021). It is well known that most gynecologic cancers (including EC, CC and OVCA) are driven by sex hormone (Baker et al., 2017), so deeply understanding the crosstalk between ferroptosis and steroid hormone signaling is helpful for tumor-targeted therapy in gynecologic cancers. In molecular mechanism, the effect of steroid hormone signaling on ferroptosis is complex (Figure 1). Firstly, steroid hormone signaling is able to regulate iron homeostasis. Secondly, abnormal steroid hormone level may affect the endogenous antioxidant capacity.

In most of gynecologic cancers, estrogen disrupts intracellular iron level and promotes free iron export into systemic circulation. Hepcidin is an important regulator of systemic iron balance. A previous study on female mouse model has reported that estrogen can reduce the expression of hepcidin (Yang et al., 2020). An *in vivo* study has reported that serum hepcidin levels declines more than 40% after E_2_ treatment in females (Lehtihet et al., 2016). Estrogen represses hepcidin expression via ERE of its promoter region; ovariectomizing reduces serum iron level in mice, but elevates the tissue iron level (Hou et al., 2012). Besides to hepcidin, estrogen also affects iron uptake and iron export in multiple gynecologic cancer types (Bajbouj et al., 2019; Riera Leal et al., 2020). Ovarian clear cell carcinoma (CCC) is a most common OVCA type. Among endometriosis-associated OVCA (EAOC), the positive percentage of ESR in CCC is only 8%, which is lower than the other OVCA types (Lai et al., 2013). In a previously study of CCC, it is found that free iron levels in endometriotic cysts and CCCs are both higher than that in nonendometriotic benign cysts (Yamaguchi et al., 2008). In doxorubicin-treated OVCA cells and BC cells, E_2_ inhibits the expression of TFRC but promotes that of ferroportin and ferritin (Bajbouj et al., 2019). In BCs, from a study of ferroptosis induced by sulfasalazine, it is reported that the expression level of TFRC in ER-positive BCs is much lower than that in the ER-negative BCs, and ESR1 knockdown increases TFRC expression (Yu et al., 2019). From a meta-analysis of CC patients in China, the result indicates that high serum iron levels may have a protective function for CC patients (Chen S. et al., 2020). In CC cell lines, the effect of E_2_ that it reduces free iron and intracellular ferritin level is only found in HaCaT cells (Riera Leal et al., 2020). However, this effect is not found in some other CC cell types (HeLa, SiHa and C33A) (Riera Leal et al., 2020). Progesterone is another important steroid sex hormone with a function of maintaining iron level, but sometimes its effect on iron regulation is contradictory. In zebrafish model, progesterone promotes the degradation of ferroportin and enhances the transcriptional expression of hepcidin (Li et al., 2016). PGRMC1, rather than the classical PRs, mediates the activity of SRC family kinases to promote hepcidin biosynthesis, and this effect can be rescued by the inhibition of SRC family kinase (Li et al., 2016). Moreover, PGRMC1 is found expressed higher in triple-negative BC (TNBC) than that in the other BC subtypes. Overexpression of *PGRMC1* reduces free iron level and inhibits ferroptosis by binding to intracellular iron; the inhibition of PGRMC1 enhances sensitivity of BC cells (MDA-MB231) to ferroptosis inducer (Zhao et al., 2023).

Abnormal steroid hormone level also affects antioxidant capacity of cancer cells so as to regulate their sensitivity to ferroptosis. For instance, IPP, an intermediate product in cholesterol synthesis, could defend ferroptosis by promoting GPX4 activity (Warner et al., 2000; Ingold et al., 2018); while inhibiting HMGCR activity by statins could also inhibit GPX4 through the same pathway (Yu et al., 2017). Moreover, increasing Sterol regulatory element-binding protein 2 (SREBP2), which promotes cholesterol synthesis by targeting HMGCR, could also suppress ferroptosis (Hong et al., 2021). Because steroid hormones (E_2_ and progesterone) have been proven to inhibit cholesterol synthesis (Metherall et al., 1996), thus, steroid hormone may act to antiferroptosis. Estrogen has been reported to protect against oxidative stress by promoting the expression of mitochondrial antioxidant enzymes (SOD2, GPXs), increasing antioxidants and reducing free radicals in many organs and cells (Irwin et al., 2008; Vina and Borras, 2010). In CC cells, E_2_ reduces NO level and MDA level (final product of the lipid peroxidation). This effect could be reversed by metformin treatment (Riera Leal et al., 2020). It can also inhibit oxidative stress through Wnt/β-catenin signaling pathway in ovarian endometrioid adenocarcinoma (Wu et al., 2007). Progesterone is reported to increase ROS level in sperm and fallopian tube fibroblasts (Gimeno-Martos et al., 2020; Wu et al., 2023). Progesterone induces ROS generation and suppresses OVCA via its receptor PR-A (Wu et al., 2017). Fallopian tube (FT) is well known as the origin of high-grade serous ovarian cancer, and defective p53 is considered as an early event in the FT epithelium-to-OVCA transition. After progesterone treatment, combining with more ROS generation, necroptosis is activated via TNF-α/RIPK1/RIPK3/MLKL pathway in p53-defective human FT fimbrial epithelial cell line (FE25 cells). The antioxidant Necrox-2 and acetylcysteine could rescue this effect (Wu et al., 2017).

Simply elevating iron level may lead to an unexpected effect. From a study of ECOA, the authors indicate that iron-induced oxidative stress may promote the production of the antioxidants, and follow by apoptosis-resistance malignant transformation of endometriosis (Kobayashi et al., 2019). A recent study also reports that persistent and mild ferroptosis increases the expression of antioxidant genes and promotes initiation of HPV-positive CC (Wang T. et al., 2022). Iron deprivation with iron chelators represses HPV E6/E7 oncogene expression and has profound antiproliferative effects in HPV-positive CC cells (HeLa and SiHa) (Braun et al., 2020). Thus, targeting ferroptosis for killing serous gynecologic cancer cells should consider their respective features (steroid hormones and the expression levels of their receptors).

### 4.3 Ferroptosis in gynecologic cancers

Ferroptosis is important for repressing the occurrence, development and metastasis of gynecologic cancers (Fan et al., 2022). In OVCA, a lot of studies have proven that enhancing lipid peroxidation is important for inhibiting OVCA cells, providing us with an emerging strategy for the OVCA treatment (Park et al., 2018; Zhao et al., 2019; Zhao X. et al., 2022). Resent study has indicated that drug-resistant OVCA cells are vulnerable to GPX4 inhibition, the ferroptosis inducer (RSL3) suppress the viability of drug-resistant OVCA cells but less affect the parental cells (Hangauer et al., 2017). Another study has proven that inhibition of monounsaturated fatty acids generation by the blockage of SCD1 sensitizes OVCA cells to RSL3 (Tesfay et al., 2019). Moreover, the inhibition of ROS generation through the Nrf2/heme oxygenase 1 (HMOX1) signaling pathway, promotes the cell proliferation of cisplatin-resistant OVCA cells (Sun et al., 2019). All the studies suggest that the drug resistance of OVCA cells depends on the endogenic antioxidant system. Ferroptosis is also proved to have a synergistic effect with chemotherapy, radiotherapy and immunotherapy in killing OVCA cells (Zhao H. et al., 2022). Ferroptosis can be induced in OVCA cells by artesunate in a ROS-dependent manner, transferrin pretreatment enhances this effect and ferrostatin-1 can rescue (Greenshields et al., 2017). Basing on the effect of ferroptosis in OVCA, a scoring system related to ferroptosis genes is used to predict the prognosis of OVCA patients (You Y. et al., 2021). Thus, there are convincing evidences to show a closely relationship of OVCA and ferroptosis.

Ferroptosis is found in SILs from patients with hrHPV infection and persistent ferroptosis contributes to an anti-ferroptosis effect in CC (Wang T. et al., 2022). In addition, more ROS generation is found in cancer cells because the cancer cells require large amount of nutrients for rapid proliferation (Wang et al., 2019). So, cancer cells increase the antioxidant activity to maintain the redox balance and prevent from cell death caused by high level of ROS. Lipid peroxidation is one of the most obvious features of ferroptosis, and its importance in CC has been proven in many studies, providing us with new insight for CC treatment by targeting ferroptosis (Jelić et al., 2018; Jelic et al., 2021).

Recently, the study on the relation between ferroptosis and EC gets many attentions. Basing on the expression profiles of cancer genomic database, some research teams have described the characteristics of ferroptosis-related genes in EC, and further provided evidences to describe the relationship between ferroptosis and the immune microenvironment, suggesting that ferroptosis-related genes could be used for the prognosis prediction of EC (Liu J. et al., 2021; Weijiao et al., 2021; Liu L. et al., 2022). A recently study has identified a centrosome microtubule-binding protein Centrosome spindle pole-associated protein (CSPP1), which functions in cell cycle-dependent cytoskeletal tissue and ciliation, to be a potential biomarker of ferroptosis, providing a novel target for the diagnosis, prognosis and therapy of EC (Wang W. et al., 2022). Another study has shown that ETS transcription factor ELK1 (ELK1) is upregulated in EC cells, and it binds to the promoter of GPX4 to antiferroptosis, indicating the ELK1/GPX4 axis might be a potential therapeutic target to develop drugs for EC (Wei et al., 2022).

Besides to gynecologic cancers, numerous studies of ferroptosis on the development and treatment of BC have been reported. And ferroptosis is considered to be a potential and valuable research direction for the treatment of BC (Sui et al., 2022). Some randomized controlled studies have reported that TF level is positively related to the incidence of ER-negative BC (Hou et al., 2021); the dietary iron supplementation is negatively correlated with the risk of BC; but in the postmenopausal women, heme iron is positively correlated with the risk of ER-positive and/or PR-positive BC (Chang et al., 2020). Iron transport protein and hepcidin have protective functions for BC patients (Pinnix et al., 2010), and the expression of TFRC is positively related to the quantity of immunocytes in BC patients (Chen et al., 2021b). Interferon -γ (IFN-γ) secreted from immunocytes suppress the cystine-uptake by reducing SLC7A11 (a ferroptosis related gene) in BC cells, followed by a lipid peroxidation and ferroptosis (Wang W. et al., 2019). In BC tissues, *SLC7A11* expresses higher than that in adjacent normal tissues. From a study of IR on BC, it is positively correlated with ESR1. ESR1 promotes SLC7A11 expression early after IR, either ESR1 or SLC7A11 knockdown enhances ferroptosis induced by IR in the ER-positive BC cells (Liu R. et al., 2021). It is reported that ESR1 inhibition enhances the sensitivity of ER-positive BC cells to ionizing radiation (IR) by inducing ferroptosis (Liu and Gu 2022). Drugs such as siramesine and lapatinib can reduce GSH level, increase ROS generation, and induce ferroptosis in BC cells (Ma et al., 2017). MUC1-C is able to interact with CD44v (CD44 variant) to stable the system xc−, increase GSH level and result in antiferroptosis in BC cells (Hasegawa et al., 2016). Notably, drug-resistant BC cells exhibits a dependence of GPX4 activity, thus, targeting GPX4 to induce ferroptosis might potentially overcome drug-resistant BC (Hangauer et al., 2017). A recently study has developed a series of small molecules that trigger to ferroptosis, and verified the effect on selectively killing drug-resistant BC stem cell-like cells (bCSC) with mesenchymal phenotypes *in vitro* (Taylor et al., 2019).

## 5 Tumor therapy in gynecologic cancers

### 5.1 Steroid hormone-targeted tumor therapy

The hormone-targeted therapy in BC can be divided into three broad categories. The first category is selective estrogen receptor modulator (SERM), which functions by binding to ER to block estrogen, including Tamoxifen, Toremifene and Fulvestrant. Tamoxifen is most commonly used in ER + BC (Legha, 1988; Yang et al., 2013), Toremifene has a comparable efficacy of Tamoxifen (Zhou et al., 2011), and Fulvestrant is the latest generation of ER inhibitor for the treatment of ER + BC (Chen P. et al., 2022). The second category is aromatase inhibitors, which functions to inhibit estrogen synthesis, including Letrozole (Mukherjee et al., 2022), Anastrozole (Nabholtz, 2006), and Exemestane (Wang Y. et al., 2022). The third category is the progesterone analogue, which functions to active PRs, including megestrol and medroxyprogesterone. Megestrol acetate (MA) is one of the first pregnancy promotors to be evaluated for hormonal treatment of advanced BC (Sedlacek, 1988). Some clinical trials have shown that luteinizing hormone releasing hormone receptor antagonists, such as Goserrelin, are also effective for BC (Moore et al., 2015).

Basing on the steroid hormone signaling characteristics of gynecological cancers, there are a lot of steroid hormone-targeted drugs applied for cancer therapy. Letrozole (an aromatase inhibitors) is used for the treatment of low-grade serous ovarian cancer and high-grade serous ovarian cancer by inhibition of estrogen generation (Heinzelmann-Schwarz et al., 2018). Besides Letrozole, there are some the third-generation aromatase inhibitors such as Anastrozole and Exemestane. Fulvestrant has a high affinity of ER and downregulates its expression. Fluvestrant is effective for in patients with disease recurrence after endocrine therapy, some ongoing clinical trials suggest that Fluvestrant may be effective in OVCA (Bidard et al., 2022; Cristofanilli et al., 2022).

Because CC is a non-hormone-responsive cancer, steroid hormone-targeted tumor therapy is not common in CC. But there are some studies which have reported the relation between long-term oral contraceptives and the increasing risk of CC (Chung et al., 2010), which reminds us to concerned about the use of hormone replacement therapy in patients with CC.

For the treatment of EC, steroid hormone-targeted drugs are usually applied in well-differentiated endometrioid adenocarcinoma, young women with early stage EC who need to maintain fertility, and patients with advanced, recurrent, or inoperable EC. Medroxyprogesterone acetate (MPA) and MA are high-potency progesterone drugs that commonly used in EC, Levonorgestrel-releasing intrauterine device (LNG-IUD) also appears to be an alternative therapy in patients who do not tolerate oral therapy (Garzon et al., 2021; Zhao H. et al., 2022; Markowska et al., 2022). The application of SERM, gonadotropin-releasing hormone agonist (GnRH agonist) (Emons and Gründker, 2021) or aromatase inhibitors is good option for the treatment of EC (Zhang et al., 2019; Markowska et al., 2022). Tamoxifen is a selective estrogen receptor modulator that is effective for low-toxicity advanced or recurrent EC (Emons et al., 2020). Some clinical trials suggest that Fluvestrant is effective for EC (Battista and Schmidt, 2016; Bogliolo et al., 2017) and other aromatase inhibitors (letrozole, anastrozole) also have meaningful efficacy in patients with recurrent EC (Slomovitz et al., 2015; Heudel et al., 2022; Slomovitz et al., 2022).

### 5.2 Ferroptosis-targeted tumor therapy

There is a good application prospect of ferroptosis induced by the blockage of endogenous antioxidant system or by the regulation of intracellular free iron level in immunotherapy, radiation therapy and drug treatment of gynecologic cancers (Table 1). For example, MA-resistant EC cells are susceptible to ferroptosis (Murakami et al., 2023); Carboplatin is effective for estrogen-resistant BC (Larsen et al., 2012). Immune checkpoint inhibitor (ICI) has made unprecedented breakthrough in some cancer types (Hernandez et al., 2022), however, due to lack of tumor-infiltrating lymphocytes, numerous cancer types with poor prognosis after ICI immunotherapy remain. A recent study has reported that CD8^+^T cells release cytokines to induce ferroptosis in OVCA cells (ID8) by suppressing system xc−, restraining cystine uptake, and enhancing lipid peroxidation (Wang K. et al., 2019). For radiation therapy (RT), there are plenty of evidences supporting its association with ferroptosis in cancers from multiple organs, including breast, ovarian, vulvar, and melanoma (Lang et al., 2019; Lei et al., 2020; Ye et al., 2020). Ionizing radiation (IR) inhibits system xc− in an ATM-dependent manner, which is a core component of DNA damage/repair systems (Matsuoka et al., 2007). In addition, Olaparib (a PARP inhibitor) can repress system xc− and induces ferroptosis in OVCA cells (Zhang et al., 2021a).

**TABLE 1:** List of ferroptosis-targeted tumor therapy drugs in gynecologic cancers.

Tumor	Drug	Mechanism of action	References
Cervical Cancer	Artemisinin	Promote free radical generation by Fe^2+^ and selectively reduces ESR1 level	Li and Zhou (2010)
	ART-conjugated phosphorescence rhenium (I) complexes	Inactivate GPX4, promote ROS generation and induce ferroptosis	Greenshields et al. (2017)
			Ye et al. (2021)
			Shield et al. (2009)
	Sorafenib	Inhibit GSH and promote ROS generation	Wang et al. (2021b)
Ovarian Cancer	Olaparib	Repress system xc− to induce ferroptosis	Zhang et al. (2021a)
	Artesunate	Promote ferritinophagy to release Fe^2+^	Du et al. (2019)
			Eling et al. (2015)
			Lin et al. (2016)
			Ooko et al. (2015)
	ART-conjugated phosphorescence rhenium (I) complexes	Inactivate GPX4, promote ROS generation and induce ferroptosis	Greenshields et al. (2017)
			Ye et al. (2021)
			Shield et al. (2009)
	Immune checkpoint inhibitors	Promote CD8^+^T cells to release cytokines that trigger ferroptosis by suppressing system xc− and enhancing lipid peroxidation	Wang et al. (2019a)
Endometrial Cancer	Quinones	Inhibit system xc−, affect iron level via the regulation of heme oxygenase and transferrin	Zhang et al. (2021b)
Breast Cancer	Sulfasalazine	Inhibit system xc− and disrupt circadian rhythms of TFRC expression	Babu and Muckenthaler (2016)
			Yoshida (2015)
			Yoshida (2018)
			Okazaki et al. (2017)
	Statins (Atorvastatin and Fluvastatin)	Inhibit GPX4 and CoQ10 through MVA pathway	Shimada et al. (2016)
			Viswanathan et al. (2017)
			Bjarnadottir et al. (2013)
			Garwood et al. (2010)
	Lapatinib (a tyrosine kinase inhibitor)	Promote ferritinophagy with autophagy-dependent manner	Ma et al. (2017)
	Vitamin C	Promote ferritinophagy to release Fe^2+^ and increase ROS level by Fenton reaction	Wang et al. (2021a)
			Singh et al. (2013)

Artemisinin is proposed to promote free radical generation by Fe^2+^ (Li and Zhou, 2010). Its safety on intravenous or intravaginal administration in patients with advanced solid tumors (NCT 02353026) and cervical intraepithelial neoplasias (NCT 02354534) have been evaluated (Chen et al., 2021c; von Hagens et al., 2017). Artesunate (ART) can promote ferritinophagy to release free iron, and thus induce ferroptosis (Eling et al., 2015; Ooko et al., 2015; Lin et al., 2016; Du et al., 2019). ART-conjugated phosphorescence rhenium (I) complexes can promote ROS generation and induce ferroptosis in OVCA and CC cells (Shield et al., 2009; Greenshields et al., 2017; Ye et al., 2021). Sorafenib is able to inhibit GSH and promote ROS generation in CC cells (Wang C. et al., 2021). Quinones inhibits cell growth of EC cells by inducing ferroptosis through both iron regulation and blockage of endogenous antioxidant system (Zhang et al., 2021b).

In addition, unsaturated fatty acid-mediated lipid peroxidation could be activated by IR, leading to ferroptosis in BC cells (MCF-7) (Lei et al., 2020). Sulfasalazine (SSZ) has recently been recognized as a system xc− inhibitor (Babu and Muckenthaler, 2016). It affects iron metabolism by disruption of circadian rhythms of TFRC expression (Yoshida, 2015; Okazaki et al., 2017; Yoshida, 2018). SSZ has been planned to be administered for clinical therapy of patients with BC and chronic pain from a Phase I clinical trial (NCT03847311). Statins can inhibit GPX4 and CoQ10 through MVA pathway, which triggers ferroptosis (Shimada et al., 2016; Viswanathan et al., 2017). Atorvastatin and fluvastatin also have antiproliferative effects on the BC cells with high expressing *HMGCR* (Garwood et al., 2010; Bjarnadottir et al., 2013). Lapatinib (a tyrosine kinase inhibitor) is reported to induce ferroptosis by activation of autophagy-dependent ferritinophagy in BC cells (Ma et al., 2017). Vitamin C can promote ferritinophagy for the release of free iron and to increase ROS level by Fenton reaction (Wang C. et al., 2021). It is reported to inhibit BC by targeting miR-93-Nrf2 axis (Singh et al., 2013).

Furthermore, steroid hormone-targeted therapy combining with ferroptosis inducer is applied in some gynecologic cancers with drug resistance. More than 30 years ago, the treatment of cisplatin, doxorubicin, cyclophosphamide, and MA is used for recurrent and metastatic EC (Hoffman et al., 1989). Besides the effect on ferroptosis, Artemisinin selectively reduces ESR1 level. The tamoxifen–artemisinin hybrids and estrogen–artemisinin hybrid compounds are highly active against BC cells (MCF-7) (Fröhlich et al., 2020).

## 6 Future perspective and conclusion

Gynecologic cancers are the most common malignant cancers in women. They affect thousands of lives and have attracted public attention due to the increased incidence rate worldwide. As a new type of RCD, ferroptosis has become a hot-spot issue in cancer research. In the past decade, there have been many studies on various aspects of ferroptosis, including molecular mechanisms, metabolic pathways, regulatory factors and tumor-related signaling pathways. Although some studies have proposed the importance of ferroptosis in gynecologic cancers, however, the underlying molecular mechanisms involved in ferroptosis and the occurrence and development of gynecological cancers have not yet been fully elucidated. We should also notice the relationship between steroid hormone signaling and ferroptosis in gynecologic cancers. The steroid hormone levels are distinct due to gynecologic cancer types, and the expression levels of steroid hormone receptors also have great difference. We examined the expression levels of steroid hormone signaling-related genes (ESR1, ESR2, PR and PGRMC1) in gynecologic cancer tissues and their adjacent samples from TCGA RNA seq data (Supplementary Figure S1). From the TCGA RNA seq data of CC with 317 patients documented, we found that ESR1 and PR were decreased in primary tumors and metastatic tumors compared to that in normal tissues, while ESR2 and PGRMC1 were increased in metastatic tumors compared to that in normal tissues and primary tumors. From the TCGA RNA seq data of OVCA with 758 patients documented, we did not find significant difference of the four genes between primary tumors and recurrent tumors. From the TCGA RNA seq data of EC with 606 patients documented, PR was decreased in primary tumors compared to that in normal tissues, while the other genes were not changed. From the TCGA RNA seq data of BC with 1,284 patients documented, ESR1 was increased in primary tumors and metastatic tumors compared to that in normal tissues, while ESR2 was decreased in primary tumors and metastatic tumors. In addition, ESR2 was found a low expression level in CC, OVCA and EC tissues. All the four genes were expressed much higher in BC tissues than that of CC, OVCA and EC tissues (Supplementary Figure S1). We next examined the expression levels of ferroptosis-related genes (GPX4, TFRC, HMGCR and ACSL4) in gynecologic cancer tissues and their adjacent samples from TCGA RNA seq data. From the TCGA RNA seq data of CC, we found that GPX4, TFRC and HMGCR were increased in primary tumors and metastatic tumors compared to that in normal tissues, while ACSL4 was decreased (Supplementary Figure S2). From the TCGA RNA seq data of OVCA, only ACSL4 was increased in recurrent tumors compared to that in primary tumors. From the TCGA RNA seq data of EC, we found that GPX4, TFRC and HMGCR were increased in primary tumors compared to that in normal tissues, while ACSL4 was not changed. From the TCGA RNA seq data of BC, only ACSL4 was decreased in primary tumors and metastatic tumors compared to that in normal tissues, while the other genes were not changed (Supplementary Figure S2).

It is important to select the best manner to trigger ferroptosis basing on the expression levels of ferroptosis-genes and characteristic of steroid hormone signaling in gynecologic cancers. In OVCA, due to the high expression of ER and low expression of PR, inhibition of estrogen signaling or activation of non-classical progesterone signaling pathway mediated by PGRMC1 are beneficial to ferroptosis. PGRMC1 has been reported to induce ferroptosis by enhancing LD lipolysis in other cell type, thus increasing unsaturated fatty acid generation to enhance lipid peroxidation and inhibiting GPX4 to reduce the antioxidant ability of OVCA cells may induce ferroptosis.

During CC development, we should also notice the function of PGRMC1 rather than ESR1 and PR. Although serotransferrin-mediated iron uptake is increased and PGRMC1 can promote LD lipolysis, but the decrease of ACSL4 may weaken this effect. The activation of the MVA pathway and the elevated GPX4 expression may constitute a strong antioxidant defense system against ferroptosis. Thus, stains and GPX4-targeted drugs may have a better treatment effect to trigger ferroptosis in CC.

In EC, due to the high expression of ESR1, PR and PGRMC1, inhibition of estrogen signaling or activation of progesterone signaling may have good effect for inducing ferroptosis. Basing on the elevated expression of TFRC and HMGCR in EC, drugs enhancing ferritinophagy and inhibiting the MVA pathway may induce ferroptosis of EC cells.

During the progress of BC development, both inhibition of estrogen signaling and enhance of progesterone signaling are necessary. Although the decrease of ACSL4 may weaken ferroptosis, enhanced LD lipolysis is still beneficial in promoting ferroptosis because the breast is rich in fat. In addition, stains and GPX4-targeted drugs may also have good treatment effect in BC. Thus, basing on the features of ferroptosis and steroid hormone signaling, the combination of steroid hormone-targeted tumor therapy and ferroptosis-targeted tumor therapy is expected to solve the problem of drug-resistance and may further enhance therapeutic efficiency of gynecologic cancers.

How to use the ferroptosis mechanism for tumor-targeted therapy still has a long way to go in gynecologic cancers. In the future, we hope to investigate the regulatory mechanism of ferroptosis on the estrogen and progesterone signaling pathways in order to provide a theoretical basis for the prevention and treatment of gynecological cancers.
