# Supplementary material for: Crosstalk between ferroptosis and steroid hormone signaling in gynecologic cancers
Source: Front Mol Biosci. 2023 Jul 4;10:1223493. doi: 10.3389/fmolb.2023.1223493 (PMC10352791; doi:10.3389/fmolb.2023.1223493)
Supplement: Supplementary file 1 [file Table1.DOCX]

***Supplementary materials***


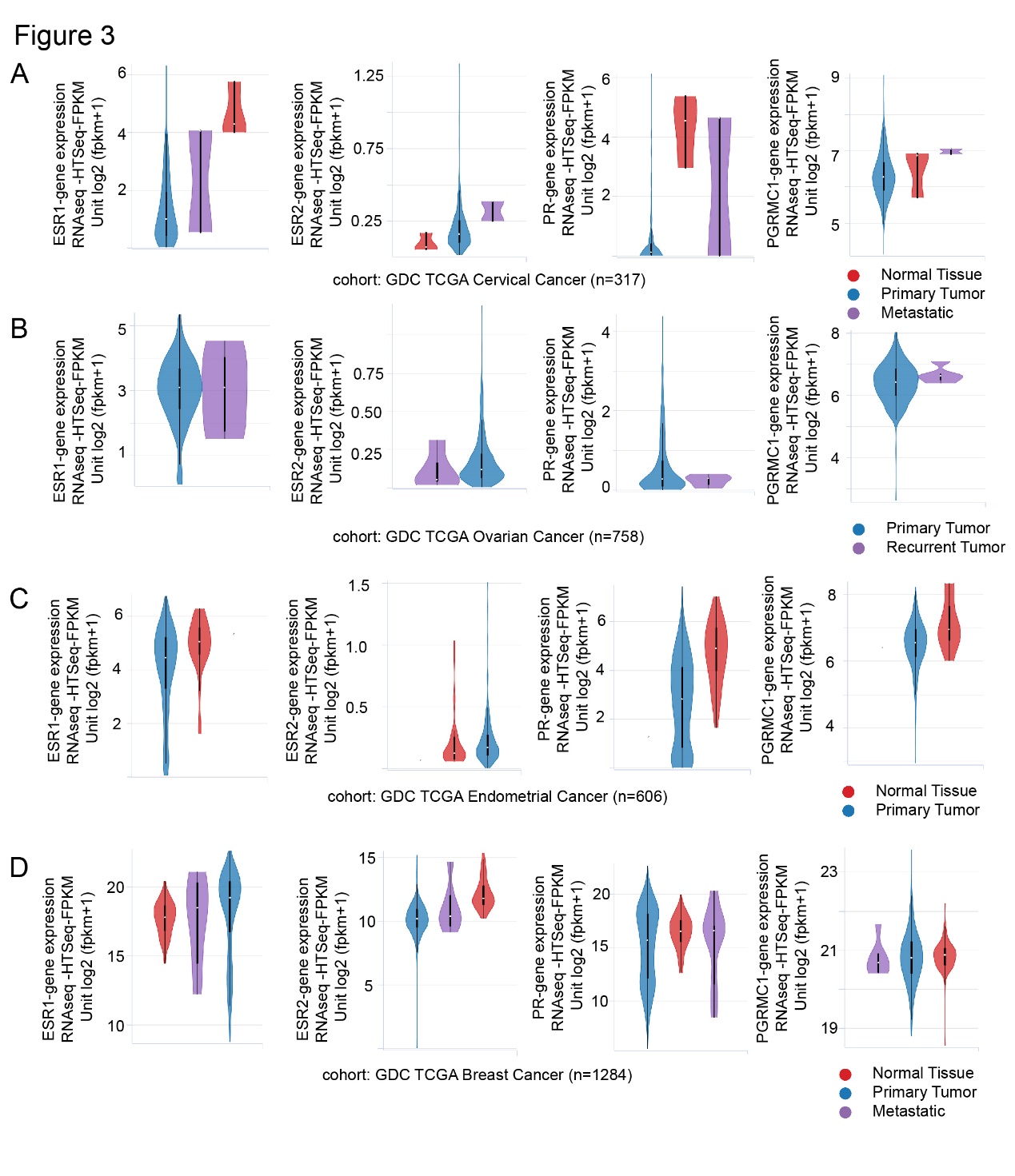


**Figure S1. The expression levels of steroid hormone receptors in gynecologic cancers.**

The expression levels of ESR1, ESR2, PR, and PGRMC1 from TCGA database in cervical cancer, ovarian cancer, endometrial cancer, and breast cancer using UCSC Xena software.


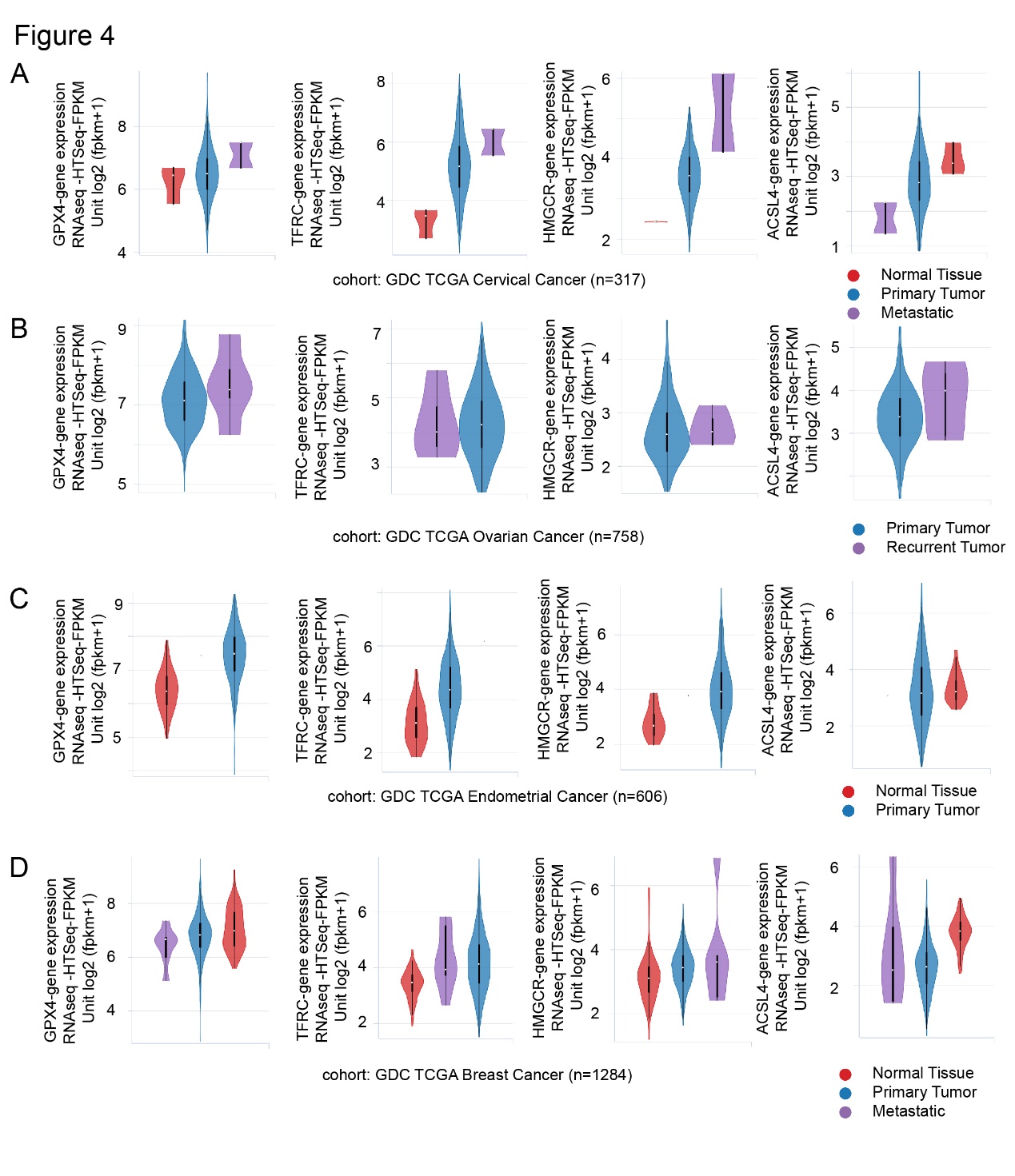


**Figure S2. The expression levels of ferroptosis-genes in gynecologic cancers.**

The expression levels of GPX4, TFRC, HMGCR, and ACSL4 from TCGA database in cervical cancer, ovarian cancer, endometrial cancer, and breast cancer using UCSC Xena software.
